# Laparoscopic-based perivascular unilateral renal sympathetic nerve denervation for treating resistant hypertension: a case report

**DOI:** 10.1038/s41440-019-0237-3

**Published:** 2019-02-27

**Authors:** Chuanyu Gao, Linwei Zhao, Lijie Zhu, Muwei Li, Degang Ding, Zhonghua Liu, Zhiqiang Fan, You Zhang, Wenli Zhao, Jiguang Wang

**Affiliations:** 1 Department of Cardiology, Zhengzhou University People’s Hospital, Zhengzhou, 450000 P.R. China; 2 Department of Cardiology, Fuwai Central China Cardiovascular Hospital, Zhengzhou, 450000 P.R. China; 3 Henan Institute of Cardiovascular Epidemiology, Zhengzhou, 450000 P.R. China; 4 Department of Urinary Surgery, Zhengzhou University People’s Hospital, Zhengzhou, 450000 P.R. China; 5 Cardiac Catheterization Room, Zhengzhou University People’s Hospital, Zhengzhou, 450000 P.R. China; 6 Department of hypertension, Shanghai Jiao Tong University Medical School Affiliated Ruijin Hospital, Shanghai, 200000 P.R. China; 7 The Shanghai Institute of Hypertension, Shanghai Jiao Tong University Medical School Affiliated Ruijin Hospital, Shanghai, 200000 P.R. China

**Keywords:** resistant hypertension, laparoscope, renal sympathetic nerve denervation

## Abstract

A 38-year-old man with a history of resistant hypertension for more than 10 years underwent laparoscopic-based perivascular unilateral renal sympathetic nerve denervation in 2012. After the operation, the patient’s blood pressure has been controllable while the antihypertensive drug intake has decreased over 6 years. Laparoscopic-based perivascular unilateral renal sympathetic nerve denervation may be a potentially feasible and effective option in treating patients with resistant hypertension.

## Introduction

Resistant hypertension is defined as blood pressure (BP) that does not remain within the normal range despite the administration of three antihypertensive medications at maximally tolerated doses, including a diuretic [1]. The sympathetic nervous system has been proven to play an important role in the progression of hypertension [1]. Catheter-based renal denervation (RDN) is a viable and safe approach that targets the renal sympathetic nerves to treat resistant hypertension. This procedure was supported by the Symplicity HTN-3 trial [2], but controversy still surrounded the issue. However, the positive findings of the SPYRAL HTN-ON MED and RADIANCE-HTN SOLO trials, published in the *Lancet* and presented at the EURO PCR in May 2018 shed considerable light on the benefits of RDN on BP controlling [3, 4]. The ablation devices used in these studies are thought to be one of the significant factors influencing the success of RDN. Since the renal sympathetic nerves are mostly located near the adventitia, but not the intima of renal arteries [5], we tried a novel method of laparoscopic-based perivascular unilateral RDN to treat resistant hypertension.

## Case summary

**Fig. 1:**
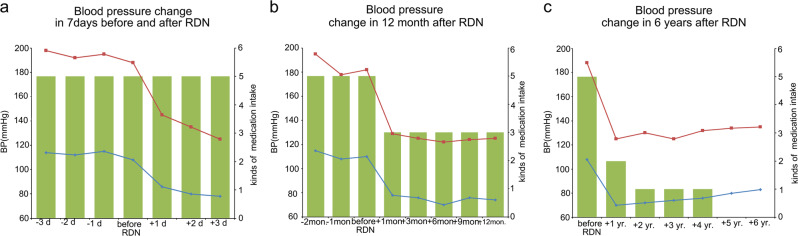
Blood pressure change before and after renal denervation (RDN). **a** Blood pressure changes in 7 days after RDN, **b** blood pressure change in 12 months after RDN, **c** blood pressure change over 6 years after RDN

In July 2012, a 38-year-old man with a history of cerebral infarctions and cerebral hemorrhages was referred to the department of cardiology for uncontrolled hypertension lasting for more than 10 years. The patient was 172 cm tall and weighed 91.5 kg. He was taking five antihypertensive drugs: felodipine 5 mg/day, valsartan 80 mg/day, metoprolol 47.5 mg/day, hydrochlorothiazide 12.5 mg/day, and spironolactone 20 mg bid. Despite the medications, his BP on examination at the hospital was 206/118 mm Hg, while the 24-h BP was 172/108 mm Hg and home BP was 195/115 mm Hg. His BP and heart rate readings at different time points are provided in Table 1 and Fig. 1. The common causes of secondary hypertension had been excluded by assessing the aldosterone to renin ratio, computed tomography scans, duplex-doppler study, metanephrine, normetanephrine, and 24-h urinary catecholamines. The patient strongly wished to gain control of his BP lowering; therefore, he agreed to undergo laparoscopic-based perivascular RDN for BP management after the nature and risks of the surgery were fully communicated to him. The patient signed an informed consent form. The surgery was approved by the ethics committee of Henan Provincial People’s Hospital (NO:2012-35).

**Table 1:** Blood pressure measurements and medications at sequential visits

Time points of visit	Home BP (mm Hg)	24-h BP monitoring (mm Hg)	Heart rate (bpm)	Body weight (kg)	Medication intake^a^
−2 months	195/115	172/108	75	91.5	Felodipine, 5 mg/day Valsartan, 80 mg/day Metoprolol, 47.5 mg/day Hydrochlorothiazide, 12.5 mg/day Spironolactone, 20 mg bid
−1 month	178/108	170/106	80	91.1	Felodipine, 5 mg/day Valsartan, 80 mg/day Metoprolol, 47.5 mg/day Hydrochlorothiazide, 25 mg/day Spironolactone, 20 mg bid
+1 day	145/86	138/80	76	90.4	As above
+2 days	135/80	–	78	–	As above
+1 month	129/78	126/76	64	90.2	Valsartan, 80 mg/day Metoprolol, 47.5 mg/day Hydrochlorothiazide, 12.5 mg/day
+3 months	125/76	128/75	65	89.0	As above
+1 year	125/70	125/73	62	90.2	As above
+2 years	130/72	130/71	65	91.7	Valsartan, 80 mg/day
+3 years	125/74	124/79	68	91.2	Valsartan, 80 mg/day
+4 years	132/76	127/76	65	90.7	Valsartan, 80 mg/day
+5 years	134/80	130/79	70	88.4	None
+6 years	135/83	132/82	68	90.6	None

+: before operation

−: after operation

*BP* blood pressure

^a^He has been taking Liputor 20 mg qd and aspirin 100 mg qd after operation

## Methods

**Fig. 2:**
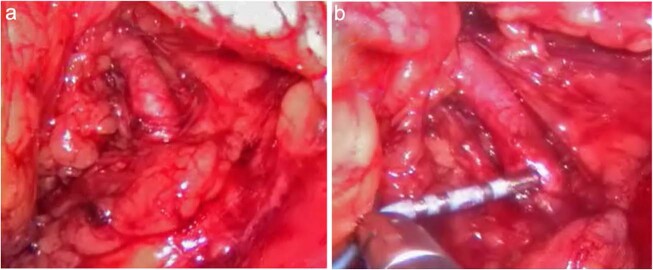
Representative image of the procedure. **a** Exposure of the renal artery, **b** ablation of the renal artery

After being anesthetized, the patient was placed in the right lateral position. A 10-mm trocar was inserted approximately 2–3 cm beneath the costal spinal angle and two 5-mm trocars were placed below the eleventh rib, approximately 3 cm laterally, followed by CO_2_ insufflation. A celioscope lens was introduced through the 10-mm trocar and two graspers were introduced through the 5-mm trocars. The homolateral renal artery was carefully separated and exposed (Fig. 2a). We used a radiofrequency (RF) ablation catheter for discrete RF ablations of 8 W for 2 min each to obtain up to six ablations that separated the renal nerves both longitudinally and rotationally from the adventitia of the renal artery (Fig. 2b).

## Results

Two months before the surgery, the patient’s home BP was 195/115 mm Hg while taking five antihypertensive medications. For his poorly controlled BP, he was asked to increase the dosage of hydrochlorothiazide from 12.5 to 25 mg/day; moreover, spironolactone 20 mg bid was added as part of the treatment by a local physician, but his BP still stabilized at approximately 180/110 mm Hg. Surprisingly, we noted that after laparoscopic-based perivascular unilateral RDN, without making any changes in his antihypertensive agent intake, his BP reduced to 145/86 mm Hg the next morning, while ambulatory BP monitoring showed that his average BP was 138/80 mm Hg. One month after the surgery, his home BP (125/78 mm Hg) and 24-h ambulatory average BP (126/76 mm Hg) decreased considerably. His antihypertensive medications were decreased to three (valsartan 80 mg/day, metoprolol 47.5 mg/day, and the hydrochlorothiazide dose was reduced from 25 to 12.5 mg/day). During the follow-ups over 6 years, the dosages of the antihypertensive medications were reduced further. After 2016, he was taking only valsartan 80 mg/day, atorvastatin 20 mg qn, and aspirin 100 mg qd. However, his BP level was relatively acceptable, as shown in Table 1 and Fig. 1. Moreover, we have not observed any significant complications in this patient during 6 years of follow-up; his body weight also stabilized at approximately 90 kg, as it was before the surgery (Table 1).

## Discussion

Surgical sympathectomy was first performed in severely hypertensive patients in the 1920s [6]. However, because of the severe side effects, including orthostatic hypotension, palpitations, anhydrosis, intestinal disturbances, loss of ejaculation, thoracic duct injuries, and atelectasis, surgery did not attain popularity [6]. Catheter-based RDN has been applied to treat resistant hypertension by ablating sympathetic nerves distributed in atrial wall from the intima of renal arteries [2]. Despite the negative results of the SYPLICITY HTN-3 study [2], the positive findings in newly published studies SPYRAL HTN-ON MED [3] and RADIANCE-HTN SOLO [4] provide some hope. Some views point out that ablating devices may be a vital aspect in determining the success of RDN. Inappropriate devices may lead to inadequate ablation, which in turn renders the effects of RDN uncertain. Whether the use of different catheters brings about different findings needs to be explored.

The primary advantage of catheter-based RDN is that it is relatively minimally invasive. However, some problems still exist. Previous studies have demonstrated that as many as 50% of the sympathetic nerve fibers may reside at depths of >3 mm from the intimal surface [7]; in other words, closer to the adventitia. The RF used during laparoscopic-based perivascular RDN is transmitted from the adventitia to the lumen, which may cause damage to more nerve fibers. We also stripped off the tissue around the renal arteries to separate and expose the renal artery, which may also destroy most of the renal nerves distributed in the adventitia. This has been confirmed in a study conducted on beagles [8, 9]. Moreover, direct stimulation using RF energy as well as the use of catheter and wire on the arterial intima may cause intimal injury and/or thrombosis [10, 11], which may trigger the progression of atherosclerosis. To minimize these side effects, we found that laparoscopic-based perivascular RDN was effective in controlling the BP and allowed for a decrease in the amount of antihypertensive drugs. No side effects were found over the 6 years of follow-up. A similar case report published in 2016 [12], presented the case of a female patient with resistant hypertension who underwent laparoscopic RDN. RF ablation was not used in her case. Her renal arteries and veins were only skeletonized circumferentially, which surprisingly led to her BP decreasing from over 220/110 mm Hg while taking three antihypertensive drugs to 100–110/70–80 mm Hg with no medications and no further complications.

In our case, in addition to stripping off the tissue around the renal artery, we applied RF ablation to the renal artery to destroy the residuary renal nerve bundles distributed deeper in the arterial wall and enhance RDN efficacy. Moreover, this was an initial attempt at laparoscopic-based perivascular RDN. Therefore, to reduce the risk of injury, we performed unilateral RDN instead of bilateral RDN, as reported in the earlier case [12]. Despite this, the BP decreased considerably.

As this is a single case, it is difficult to exclude the effect of other internal and external factors on the BP. After surgery, some inadvertent changes in the lifestyle that may lower the BP may have occurred. Clarifying the effects of these factors would require larger sample sizes and controls. As an initial attempt, based on this case report, we can successfully state that laparoscopic-based perivascular unilateral RDN is a potentially feasible and effective option for treating resistant hypertension, although additional clinical and animal studies are required.
